# Investigation of 3D choroidal components in myopic populations using ultra-widefield OCTA

**DOI:** 10.1038/s41433-025-04203-4

**Published:** 2026-01-13

**Authors:** Tengbo Rao, Jiarui Yang, Yanfeng Liao, Yi Ding, Yiwen Shi, Huijin Chen, Xuemin Li

**Affiliations:** 1 Department of Ophthalmology, Peking University Third Hospital, Beijing, China; 2 Beijing Key Laboratory of Restoration of Damaged Ocular Nerve, Peking University Third Hospital, Beijing, China

**Keywords:** Eye manifestations, Outcomes research

## Abstract

**Purpose:**

This study investigates changes in vascular and stromal components of choroidal vascular layers using ultra-widefield optical coherence tomography angiography (OCTA).

**Methods:**

A cross-sectional study included 147 participants with varying myopia degrees, categorised as low myopia (70 eyes), moderate myopia (104 eyes), and high myopia (110 eyes) based on refractive status. The TowardPi Wide-field OCTA measured choroidal parameters, including thickness, choriocapillaris density, vascular volume, and stromal volume. Differences in these parameters were analysed among the three myopia groups.

**Results:**

Choroidal thickness decreased with increasing myopia, notably at the sub-fovea (LM vs MM vs HM: 297.56 ± 83.19 μm vs 230.13 ± 77.35 μm vs 190.49 ± 70.24 μm, P < 0.001) and macular region (258.20 ± 67.40 μm vs 215.37 ± 56.07 μm vs 186.01 ± 49.10 μm, P < 0.001). Choriocapillaris density in the macular region increased with myopia severity (48.06 ± 1.36% vs 48.15 ± 0.99% vs 48.74 ± 1.11%, P < 0.001). Vascular volume declined most in the macular (LM vs MM: 5.98 ± 1.62 mm³ vs 4.21 ± 1.52 mm³, P < 0.001). Stromal volume changes were prominent between moderate and high myopia (MM vs HM: 6.59 ± 1.48 mm³ vs 6.12 ± 0.75 mm³, P < 0.001). Axial length correlated with stromal volume (R = -0.498, P < 0.001), while spherical equivalent correlated with choroidal volume (R = 0.474, P < 0.001).

**Conclusion:**

Wide-field OCTA effectively highlights choroidal component changes associated with myopia severity, offering insights into its structural alterations.

## Introduction

Myopia is one of the most common eye conditions worldwide, with a prevalence ranging from 10 to 30% in the adult population in many countries [1]. In some parts of East and Southeast Asia, the prevalence can reach 80–90% among young adults [2]. The choroid, as the primary vascular layer of the eye, also plays a crucial role in the onset and development of myopia [3]. The most severe complication of myopia, the occurrence of choroidal neovascularisation, is closely related to changes in choroidal vasculature [4]. Therefore, exploring changes in choroidal vascular morphology is crucial for understanding the mechanisms underlying the onset and progression of myopia.

In recent years, OCTA has played a crucial role in the study of the choroid [5]. As a rapid and accurate examination technique, SS-OCTA was previously primarily used to scan within a 6 × 6 mm range centred on the macula [6]. Through this examination method, previous studies have found that as the severity of myopia increases, the thickness of the choroid in the macular region significantly decreases. However, the visibility of the deeper choroid remains insufficient and needs improvement [7]. Meanwhile, localised OCTA scans cannot fully reflect the overall retinal and choroidal blood flow changes.

With technological advancements, ultra-widefield SS-OCTA has entered clinical practice, providing more comprehensive choroidal vascular information through digitalisation [8]. Previous studies utilising wide-field OCTA technology have found that in myopic patients, choroidal thinning is primarily concentrated in the macular region [9]. Additionally, as moderate myopia progresses to high myopia, significant changes occur in the retinal and choroidal structures, as well as in vascular density [10]. However, current wide-field OCTA studies on myopia primarily focus on conventional parameters such as changes in choroidal thickness and vascular density, with relatively few studies examining the role of changes in different grids of choroidal blood volume in myopia.

In this study, we aim to use wide-field OCTA technology to analyse the volumetric changes in the vascular and stromal components of the choroid in individuals with varying degrees of myopia. Our study might provide insight into the pathogenesis of myopia based on the characteristics of choroidal vascular networks in different choroidal components.

## Materials and methods

### Participants

This study was conducted at the Peking University Third Hospital. This study was approved by the institutional committee on medical ethics and adhered to the tenets of the Declaration of Helsinki. The ocular data of the volunteers were used with their consent. The volunteers in this study comprised a healthy population with varying degrees of myopia. Both eyes of the volunteers were included in the study. All volunteers underwent comprehensive ophthalmologic examinations, which included assessments of best-corrected visual acuity (BCVA), refraction, axial length, and wide-field SS-OCTA. The exclusion criteria were as follows: (1) previously diagnosed systemic diseases; (2) other ocular diseases, such as cataract, glaucoma, or retinal diseases; (3) history of intraocular surgery, laser surgery, or refractive surgery; (4) ocular trauma; (5) retinal choroidal atrophy or those identified with lesions affecting OCTA signals, such as myopia-related neovascularisation. The dioptres (D) were collected and converted to spherical equivalent (SE), which was the spherical dioptric power plus half of the cylindrical dioptric power. The IOLMaster 700 (Carl Zeiss Meditec, Jena, Germany) was used to measure axial length. In this study, patients were categorised based on their refractive error as follows: low myopia (LM; refractive error with SE ≥ −3.00 D), moderate myopia (MM; refractive error of −6.0 D < SE ≤ −3.0D), or high myopia (HM; refractive error with SE ≤ −6 D).

### Ultra-widefield SS-OCTA

All participants were imaged using a 400-kHz SS-OCTA (TowardPi BMizar; TowardPi Medical Technology, Beijing, China) capable of performing 400,000 scans per second. This device employs a swept-source vertical-cavity surface-emitting laser (VCSEL) with a wavelength of 1060 nm, providing a transverse resolution of 10 μm and an in-depth (optical) resolution of 3.8 μm in tissue. Each OCT volume was composed of 2560 pixels in depth, 1536 pixels in width, and 1280 B-scans, corresponding to nominal physical dimensions of 6 mm in depth, 24 mm in width, and 20 mm. The OCTA system equipped with an integrated eye-tracking rate of 128 Hz, which enables the processing of eye motion and blinking. Additionally, all images included in this study were automatically graded by the system, with only those scoring above 9 (on a scale of 10) being included for analysis.

Intra-session and inter-session repeatability testing of wide-field OCTA has been conducted in several previous studies [9, 11]. Therefore, repeated OCTA measurements were not performed on the participants in this study.

### Choroidal segmentation and layering method with image processing

**Fig. 1:**
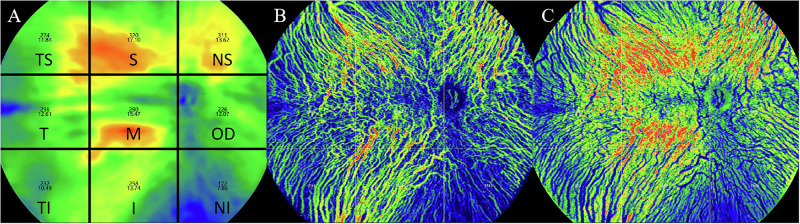
Methods for choroidal segmentation and relevant parameter measurements. **A** The choroidal thickness distribution map and the method for regional segmentation are illustrated as follows. The 24 × 20 mm choroidal thickness map is divided into a 3 × 3 grid, consisting of the following regions: tempo-superior (TS), superior (S), nasal-superior (NS), tempo (T), macular (M), optic disc (OD), tempo-inferior (TI), inferior (I), and nasal-inferior (NI); **B** Choroidal Vascular Volume Distribution Map; **C** Choroidal Stromal Volume Distribution Map.

The extraction of three-dimensional choroidal data in this study relied on measurements obtained using the system’s built-in software. The choroidal vessels were divided by the system’s built-in segmentation software into the choriocapillaris layer (from Bruch’s membrane to 29 μm below Bruch’s membrane) and the large and medium choroidal vessel layer (from 29 μm below Bruch’s membrane to the choroidal-scleral interface) based on the diameter of the blood vessels. The segmentation results of each OCTA image were manually verified, and any images with segmentation errors were corrected manually. Following the methodology of previous research, we divided the whole choroid zone into a 3 × 3 grid (comprised of nine rectangles: tempo-superior (TS), superior (S), nasal-superior (NS), tempo (T), macular (M), optic disc (OD), tempo-inferior (TI), inferior (I), and nasal-inferior (NI)) for analysis [12] (Fig. 1).

### Choroidal thickness and choroidal volume measurement

This study measured choroidal thickness from multiple perspectives, including the average wide-field choroidal thickness (WF-CT), the average choroidal thickness (CT) in each choroidal grid, the sub-fovea choroidal thickness (SFCT). WF-CT, CT, and SFCT were obtained through automated measurements by the machine.

In this study, the system’s built-in software was used to measure the following parameters of 29 μm below the Bruch’s membrane to the choroid-sclera interface (CSI): choroidal Volume (CV), choroidal vascular volume (CVV), choroidal stromal volume (CSV), and choroidal vascular index (CVI), both under wide-field and within each 3 × 3 grid (Fig. 1). The choroidal volume parameters were reconstructed based on 1280 B-scan images captured by wide-field OCTA. CVI is defined as the ratio of choroidal vascular volume to the total volume within a given region.

### Statistical analyses

Statistical analyses were conducted using the SPSS software (version 26.0; IBM). Categorical data were presented as frequency counts, and the values of all continuous variables were presented as mean ± standard deviation. Various statistical methods were applied to compare differences in patient characteristics. For samples larger than 50, the Kolmogorov-Smirnov test is used to assess normality, while for samples smaller than 50, the Shapiro-Wilk test is applied to test for normality. One-Way ANOVA Analysis was used to compare data that conformed to normality, whereas the nonparametric test was used to compare data that did not conform to normality. The relationship between axial length and choroidal vascular parameters was analysed using a linear regression model. A priori power analysis was conducted using G*Power software, assuming a medium effect size (Cohen’s f = 0.25), a significance level of 0.05, and a desired power of 0.80. The analysis indicated that approximately 30 eyes per group would be required, resulting in a total sample size of 90 eyes. *P* value < 0.05 was regarded as statistically significant.

## Result

This study included 147 volunteers, with a total of 284 eyes: 70 eyes with low myopia, 104 eyes with moderate myopia, and 110 eyes with high myopia. Among them, 74 eyes underwent axial length measurements. The baseline characteristics of the volunteers are listed in Table 1.

**Table 1:** Demographic and clinical characteristics of eyes included in the study.

		LM	MM	HM	*P*
Eyes, n		70	104	110	/
Age (y)		30.53 ± 8.37	31.13 ± 10.48	28.96 ± 6.47	0.167
Gender	Male, n	33	37	40	0.249
	Female, n	37	67	70	
Axial length (mm)		24.69 ± 1.43	25.54 ± 0.99	26.70 ± 0.63	<0.001***
SE (D)		–1.18 ± 1.10	–4.36 ± 0.89	–7.65 ± 1.37	<0.001***

***P < 0.001.

*n* number of eyes, *LM* low myopia, *MM* moderate myopia, *HM* high myopia.

### The relationship between choroidal thickness metrics and the degree of myopia

Figure S1 and Table S1 show the differences in choroidal thickness among individuals with varying degrees of myopia. For different choroidal regions, the choroidal thickness gradually decreased with increasing myopia severity. This trend was most notable in the WF-CT (LM vs MM vs HM, 204.27 ± 41.23 μm vs 185.74 ± 32.44 μm vs 171.97 ± 29.47 μm, *P* < 0.001), the SF-CT (LM vs MM vs HM, 297.56 ± 83.19 μm vs 230.13 ± 77.35 μm vs 190.49 ± 70.24 μm, *P* < 0.001), and M-CT (LM vs MM vs HM, 258.20 ± 67.40 μm vs 215.37 ± 56.07 μm vs 186.01 ± 49.10 μm, *P* < 0.001). In the superior and temporal regions, as well as the nasal-inferior region, choroidal thickness changes between moderate and high myopia were not significant. However, in the NS and TI regions, changes in choroidal thickness between low and high myopia were not significant. The results above indicate that changes in choroidal thickness across different regions are associated with the stages of myopia progression.

### Relationship between choriocapillaris density and degree of myopia

Figure S2 and Table S2 show the trend of changes in the choroidal capillaries among eyes with varying degrees of myopia. The changes in capillary density within different choroidal regions are not significant. We found that only in the macular region, the blood flow density of the choroidal capillary layer gradually increased with the progression of myopia. (LM vs MM vs HM, 48.06 ± 1.36% vs 48.15 ± 0.99% vs 48.74 ± 1.11%, *P* < 0.001) This suggests that the impact of myopia on the choroidal capillary layer is primarily concentrated in the macular region, with no significant changes observed in other areas.

### Relationship between choroidal volume and degree of myopia

Figure S3 and Table S3 illustrate the trend of choroidal volume changes with varying degrees of myopia. The trend in choroidal volume changes aligns closely with that of choroidal thickness, both of which decrease as the degree of myopia increases. The changes in choroidal volume are also most notable in the macular region. (LM vs MM vs HM, 13.24 ± 4.21 mm^3^ vs 10.98 ± 3.01 mm^3^ vs 8.81 ± 2.97  mm^3^, *P* < 0.001) In the intergroup comparison, differences between moderate and high myopia were found in each choroidal region. However, volume differences between low and moderate myopia were only observed in the I-CV, NI-CV and M-CV. In other regions, although a consistent trend was observed, no significant differences were detected. This may suggest that early myopic changes could exhibit heterogeneity across different choroidal regions.

### Relationship between choroidal components and degree of myopia

**Fig. 2:**
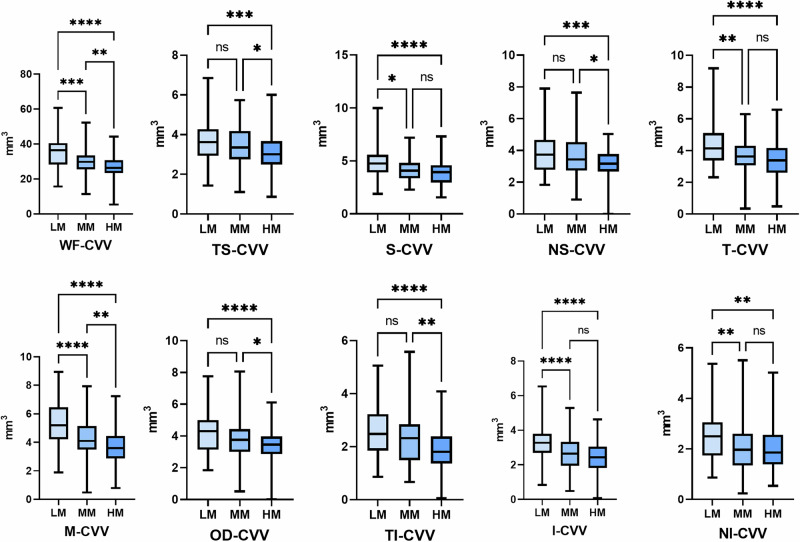
Comparison of choroidal vascular volume in different degrees of myopia. LM low myopia, MM moderate myopia, HM high myopia, CVV choroidal vascular volume, *P < 0.05, **P < 0.01, ***P < 0.001, ****P < 0.0001.

**Fig. 3:**
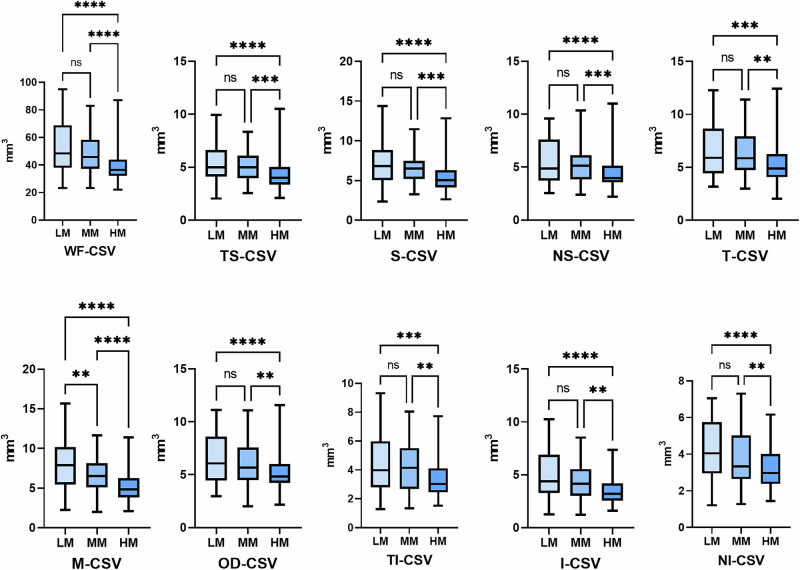
Comparison of choroidal stroma volume in different degrees of myopia. LM low myopia, MM moderate myopia, HM high myopia, CSV choroidal stroma volume, *P < 0.05, **P < 0.01, ***P < 0.001, ****P < 0.0001.

Figures 2, 3, and S4 respectively show the trend of changes in different choroidal components across groups with varying degrees of myopia. Both CVV and CSV show a decreasing trend across different choroidal regions. The changes in CVV are primarily observed between the low myopia and moderate myopia groups, with the most significant differences in M-CVV (LM vs MM, 5.98 ± 1.62 mm^3^ vs 4.21 ± 1.52 mm^3^, *P* < 0.001), T-CVV (LM vs MM, 4.65 ± 9.48 mm^3^ vs 3.56 ± 1.18 mm^3^, *P* < 0.001), and I-CVV (LM vs MM, 3.63 ± 0.89 mm^3^ vs 2.55 ± 1.49 mm^3^, *P* < 0.001). The changes in the choroidal stroma are primarily concentrated between the moderate and high myopia groups, with alterations in CSV observed only in the macular region between the low myopia and moderate myopia groups. This may suggest that changes in the choroidal stroma may primarily occur during the transition from moderate to high myopia.

In addition, we also analysed the changes in CVI across different regions. Due to the simultaneous decrease in both the vascular and stromal volumes of the choroid, we found that the CVI does not change significantly during the transition from mild myopia to moderate myopia, or it shows only a slight downward trend. As the degree of myopia gradually increases, CSV rapidly decreases, leading to an upward trend in CVI from moderate myopia to high myopia. This suggests that during the transition from moderate myopia to high myopia, the atrophy of the choroidal stromal volume plays an important role in the development and progression of myopia.

### The relationship between axial length and choroidal parameters

To assess which choroidal parameters align most consistently with changes in axial length and spherical equivalent, Table 2 analyses the correlations between the indicators. We found that for axial length, the highest correlation coefficients were observed with the average choroidal thickness in the macular region (R = –0.438, *P* < 0.001) and the choroidal stromal volume in the macular region (R = –0.498, *P* < 0.001). The spherical equivalent showed better correlations with many choroidal parameters, especially those in the macular region. The correlation of CVI with both indicators is relatively low, suggesting that CVI may have limitations in reflecting changes in the degree of myopia.

**Table 2:** Axial length and choroidal parameter correlation analysis.

		WF-CT	M-CT	WF-CCD	M-CCD	WF-CV	M-CV	WF-CVV	M-CVV	WF-CSV	M-CSV	WF-CVI	M-CVI
AL	R	–0.404	–0.438	–0.008	0.369	–0.409	–0.439	–0.385	–0.359	-0.426	–0.498	–0.24	–0.029
	P	<0.001***	<0.001***	0.947	0.001**	<0.001***	<0.001***	0.001***	0.002**	<0.001***	<0.001***	0.04*	0.806
SE	R	0.382	0.463	0.041	-0.228	0.394	0.474	0.398	0.458	0.333	0.453	–0.022	–0.086
	P	<0.001***	<0.001***	0.495	<0.001***	<0.001***	<0.001***	<0.001***	<0.001***	<0.001***	<0.001***	0.707	0.148

*P < 0.05, **P < 0.01, and ***P < 0.001.

*AL* axial length, *WF* wide field, *M* macular, *CVI* choroidal vascular index, *CV* choroidal volume, *CVV* choroidal vascular volume, *CSV* choroidal stroma volume.

## Discussion

OCTA has played an important role in clinical practice in examining retinal and choroidal vascular characteristics [13–15]. As a relatively new technology, ultra-widefield OCTA offers a broader field of view compared to traditional OCTA, and it has already been applied to assess retinal and choroidal changes in various diseases [16]. In our study, we explored the variations in a range of wide-field OCTA choroidal parameters across different myopia groups. We found that choroidal thickness decreased with increasing myopia severity, with this trend being most prominent in WF-CT, SF-CT, and M-CT. Additionally, we analysed the density trend of the choriocapillaris layer and found that its density increased with the severity of myopia only in the macular region. Subsequently, we analysed the differences in choroidal components across different degrees of myopia from a volumetric perspective. We found that changes in CSV were mainly observed between the moderate and high myopia groups. Moreover, the changes in CVV across different stages of myopia exhibit regional heterogeneity. The CVI calculated from volume measurements showed an increasing trend between the moderate and high myopia groups.

It is widely accepted that choroidal thinning is an important indicator of the progression of myopia [17, 18]. Choroidal thickness measurements were primarily performed on B-scan imaging using image segmentation techniques, focusing on restricted areas. Prior studies have found that, the choroid is thinnest on the nasal side in myopic individuals [19]. Subsequent studies utilising wide-field OCT support the former results that choroidal thinning primarily occurs in the macular region, with additional thinning occurring in the inferior and nasal regions of the entire fundus outside the macula [20]. However, CT measurements based on B-scan showed great variability since the bias on the selection of measured point, which has made it challenging to characterise the overall trend in choroidal thickness changes. Our study comprehensively examined the relationship between CT and myopia across different regions and the entire wide-field view. We first observed that the thinnest point of choroidal thickness for low, moderate, and high myopia occurred in the nasal-inferior, inferior, and temporal-inferior regions, respectively (Supplementary table 2). However, there was no significant difference between the moderate and high myopia groups, which is consistent with previous reports in the literature [9]. This suggests that the changes of thickness in the choroid caused by myopia are most prominent during the initial progression of myopia, while these changes become less evident as myopia severity increases.

The choroidal capillary layer is one of the key vascular layers in the future progression of high myopia to pathological myopia. Some past studies have found that in high myopia, the density of the choroidal capillary layer may not show significant changes or may exhibit a downward trend [21, 22]. A recent study found that the density of the choroidal capillary layer increases in high myopia, which is consistent with our findings [10]. Furthermore, this change is confined to the macular region, while in other choroidal regions, the density of the choroidal capillaries does not change with increasing myopia. In the early stages of high myopia, the choroidal capillary density may increase to address ischaemia and hypoxia in deeper retinal structures. This change may be a precursor to the development of myopia-related choroidal neovascularisation in some cases of highly myopic eyes. It also suggests why myopia-related choroidal neovascularisation is more likely to occur in the macular region [23]. Further exploration of the molecular mechanisms could provide additional validation for this hypothesis.

The advent of wide-field OCTA has made it possible to analyse volumetric data across different regions. Quadrant-specific changes in choroidal components can provide deeper insights into the volumetric distribution of choroidal vessels. Previous studies on choroidal volume have primarily focused on the 6 × 6 mm area of the macular region, and the results indicate that foveal and nasal quadrants are important for monitoring choroidal changes in high myopia [24, 25], which is in accordance with the trend of thickness changes. However, the scanning area is relatively small, which could not fully represent the characteristics of choroid. Our study used wide-field OCTA to explore changes in the choroidal components in the peripheral regions during the progression of myopia.

We found that with the increase in the degree of myopia, both CVV and CSV exhibited a declining trend, which is consistent with previous literature reports [22, 24]. However, the changes in different choroidal components vary across regions, with the differences being most pronounced in the wide-field and macular areas, while other regions exhibit heterogeneity. Previous studies have also found that changes in CVV are most pronounced in the macular region, which is consistent with our conclusions [26]. Additionally, we observed that the changes in CSV in different choroidal regions mainly occurred during the transition from moderate to high myopia, rather than between low and high myopia. Therefore, we believe that stromal atrophy in peripheral regions may serve as an imaging marker for distinguishing between moderate and high myopia. The choroidal stroma plays a crucial role in the mechanical stability of the eyeball [27, 28]. Atrophy primarily occurring in the choroidal stroma may be one of the key factors in the development of scleral staphyloma in eyes with high myopia.

Another point we observed is that the trend of CVI changes in relation to myopia may vary depending on the calculation method used. In previous studies based on OCT B-scan results, a method comparing the luminal area and stroma area through binarisation was used, and it was found that CVI tends to decrease as the degree of myopia increases [29, 30]. However, the impact of myopia on 3D-CVI remains inconclusive. When calculating the 3D-CVI, due to the more significant decrease in CSV compared to CVV, we found that the CVI exhibited an upward trend during the progression from moderate to high myopia. This finding is consistent with previous studies that calculated 3D-CVI [26]. In another study, 3D-CVI was found to be significantly lower in the high myopia group compared to the low and moderate myopia groups [24]. We believe the observed differences between 2D-CVI and 3D-CVI might originate from their distinct calculation methods. The traditional area-based method for calculating CVI, which relies on B-scan results, is relatively limited to scans passing through the macular region. It is possible that the reduction in CVV at the fovea is most prominent, leading to a decrease in CVI when using this approach. Additionally, CVI calculated using the binarisation method may be affected by the blooming effect. When high signal amplification is used, the image appears brighter, and the number of white pixels increases; conversely, with lower amplification settings, the image becomes darker and the white pixels decrease [31]. In contrast, 3D-CVI, calculated by overlaying blood flow signals, is less influenced by image contrast than binarisation methods, making its results potentially more objective and accurate. This also highlights that different methods may lead to variations in CVI calculations.

Finally, we analysed the correlation between AL and SE with choroidal volume parameters. Previous studies have measured the correlation between the macular CVI and axial length using various methods. One approach involved measuring the 2D CVI based on B-scan, resulting in a correlation coefficient of –0.259 [32]. Another method measured the 3D CVI within a 1 mm circle centred on the fovea, yielding a correlation coefficient of –0.289 [24]. We found that axial length showed a low degree of correlation with most choroidal parameters, but it had a poor correlation with the CVI in the macular region. The measured macular CVI value was obtained from the central 1/9 portion of a 24 × 20 mm wide-angle OCTA scan. For the entire wide-angle region, the correlation coefficient of axial length and the CVI within the 24 × 20 mm area of the choroid was –0.24. This may be due to the wide-field OCTA examination, which allows for the assessment of a broader range of choroidal changes. As myopia progresses from high myopia to pathological myopia, characteristic features such as retinoschisis or posterior scleral staphyloma often occur in the peripheral retina and choroid [33]. These changes can be easily overlooked if attention is limited to the macular region. Therefore, wide-field OCTA imaging is of great significance for revealing comprehensive myopia-related choroidal changes.

The limitations of this study primarily involve the following aspects. Firstly, in the design of this study, not all enroled patients underwent axial length measurements. The OCTA parameters may exhibit slight deviations without axial length correction. Although such deviations may not affect the conclusions or the data distribution, from a rigorous perspective, axial length correction should ideally be applied to the OCTA data. In addition, during the application of ultra-widefield OCTA, certain pixel-based parameters, such as choriocapillaris density, may be affected by reduced resolution, potentially compromising data accuracy. Future studies may incorporate localised high-resolution OCTA scans to improve measurement precision. Moreover, this study did not perform repeated measurements on the patients, which may introduce random errors caused by the operator. Finally, since this study focuses on myopia without significant retinal pathology, future research could explore the relationship between high myopia and conditions such as retinoschisis or myopic choroidal neovascularisation, as well as their impact on choroidal structural parameters.

## Conclusion

This study utilised wide-field OCTA to investigate the trends of changes in different choroidal components and regions during the progression of myopia. We observed regional heterogeneity in changes in CVV with increasing myopia severity, while changes in CSV were primarily evident between moderate and high myopia. Moreover, traditional CVI may still have limitations in understanding the progression of myopia. In summary, this study highlights and underscores the critical role of wide-field OCTA in uncovering the processes underlying the development and progression of myopia and other diseases.

## Summary

### What was known before

- Relationship Between Choroidal Thickness and Myopia: Previous studies have shown that choroidal thickness decreases with the progression of myopia, particularly in the macular region and sub-foveal areas.
- Changes in Choroidal Microcirculation: Research indicates alterations in the choriocapillaris density in myopic eyes, but there remains controversy regarding the regional differences and specific patterns.
- Vascular and Stromal Components of the Choroid: Some studies using optical coherence tomography (OCT) have analysed choroidal vascular and stromal volumes, but these analyses are often limited to localised areas, such as the macular region, with less emphasis on the entire choroid.

### What this study adds

- Layered Analysis of Vascular and Stromal Components: Unlike previous studies that primarily focused on choroidal thickness, this research provides a detailed, layered quantitative analysis of the vascular and stromal components of the choroid.
- Comprehensive Assessment Across Wide Areas: The study evaluates multiple regions of the choroid, including the macular region, inferior areas, and peripheral zones, offering a more holistic perspective.
- Identification of Distinct Patterns in Myopia Progression: By comparing changes in choroidal parameters across low, moderate, and high myopia groups, this study uncovers specific patterns of vascular and stromal alterations and their potential mechanisms during myopia progression.

## Supplementary information

Supplementary Material

## Data Availability

The datasets analysed during the current study are available from the corresponding author on reasonable request.
