# Supplementary material for: Investigation of 3D choroidal components in myopic populations using ultra-widefield OCTA
Source: Eye (Lond). 2026 Jan 13;40(5):630–6. doi: 10.1038/s41433-025-04203-4 (PMC13013728; doi:10.1038/s41433-025-04203-4)
Supplement: Supplementary file 1 — Supplementary Material [file 41433_2025_4203_MOESM1_ESM.docx]

**Supplementary Table**

**Table S1. The distribution of choroidal thickness in people with different degrees of myopia**

| (μm) | LM | MM | HM | *P* |
| --- | --- | --- | --- | --- |
| WF-CT | 204.27 ± 41.23 | 185.74 ± 32.44 | 171.97 ± 29.47 | <0.001*** |
| SFCT | 297.56 ± 83.19 | 230.13 ± 77.35 | 190.49 ± 70.24 | <0.001*** |
| Tempo-superior | 221.44 ± 55.43 | 207.19 ± 51.13 | 193.54 ± 44.04 | 0.001** |
| Superior | 226.40 ± 58.19 | 208.17 ± 45.50 | 194.20 ± 45.60 | <0.001*** |
| Nasal-superior | 217.69 ± 54.03 | 209.83 ± 51.06 | 192.23 ± 37.92 | 0.001** |
| Tempo | 211.20 ± 50.23 | 194.17 ± 40.46 | 183.48 ± 41.33 | <0.001*** |
| Macular | 258.20 ± 67.40 | 215.37 ± 56.07 | 186.01 ± 49.10 | <0.001*** |
| Optic disc | 211.64 ± 53.26 | 195.86 ± 44.62 | 183.85 ± 38.69 | <0.001*** |
| Tempo-inferior | 159.11 ± 42.18 | 148.84 ± 42.69 | 135.48 ± 33.28 | <0.001*** |
| Inferior | 165.19 ± 47.26 | 142.33 ± 38.63 | 133.73 ± 32.81 | <0.001*** |
| Nasal-inferior | 159.86 ± 44.45 | 143.90 ± 41.28 | 140.15 ± 35.08 | 0.004** |

Note: LM = Low Myopia, MM = Moderate Myopia, HM = High Myopia, CT = Choroidal Thickness, WF = Wide-Field, SF = Sub-fovea, **P*<0.05, ***P*<0.01, and ****P*<0.001.

**Table S2. The distribution of choroidal capillary density in people with different degrees of myopia**

| CCD（%） | LM | MM | HM | P |
| --- | --- | --- | --- | --- |
| WF | 47.01 ± 1.45 | 46.88 ± 1.75 | 46.96 ± 1.61 | 0.846 |
| Tempo-superior | 50.19 ± 3.26 | 48.76 ± 5.76 | 49.57 ± 3.30 | 0.098 |
| Superior | 49.59 ± 1.35 | 49.24 ± 1.30 | 49.31 ± 1.21 | 0.201 |
| Nasal-superior | 48.76 ± 3.34 | 47.99 ± 4.10 | 47.81 ± 3.79 | 0.245 |
| Tempo | 46.99 ± 2.51 | 47.15 ± 2.49 | 47.31 ± 2.65 | 0.713 |
| Macular | 48.06 ± 1.36 | 48.15 ± 0.99 | 48.74 ± 1.11 | <0.001*** |
| Optic disc | 46.43 ± 3.08 | 45.88 ± 3.11 | 46.12 ± 2.85 | 0.505 |
| Tempo-inferior | 42.41 ± 4.99 | 43.25 ± 5.26 | 42.61 ± 5.05 | 0.509 |
| Inferior | 47.04 ± 2.98 | 47.55 ± 3.08 | 47.64 ± 2.70 | 0.379 |
| Nasal-inferior | 43.76 ± 4.79 | 43.86 ± 6.40 | 43.60 ± 5.55 | 0.945 |

Note: LM = Low Myopia, MM = Moderate Myopia, HM = High Myopia, CCD = Choroidal Capillary Density, WF = Wide-Field, **P*<0.05, ***P*<0.01, and ****P*<0.001.

**Table S3. The distribution of choroidal metrics in people with different degrees of myopia**

| Parameters | LM | MM | HM | *P* |
| --- | --- | --- | --- | --- |
| WF choroidal vascular parameters | | | |  |
| CV (mm^3^) | 86.44 ± 24.73 | 78.17 ± 16.96 | 67.07 ± 15.64 | <0.001*** |
| CVV (×10^6^μm^3^) | 37741.13 ± 7767.55 | 29507.10 ± 8850.56 | 27369.50 ± 8613.87 | <0.001*** |
| CSV (×10^6^μm^3^) | 71541.50 ± 7582.94 | 65899.28 ± 14788.13 | 61221.75 ± 7548.20 | 0.026* |
| CVI | 0.34 ± 0.03 | 0.30 ± 0.03 | 0.30 ± 0.04 | <0.001*** |
| CV (mm^3^) by grid | | | | |
| Tempo-superior | 8.98 ± 2.58 | 8.57 ± 1.95 | 7.39 ± 2.03 | <0.001*** |
| Superior | 11.55 ± 3.83 | 10.58 ± 2.59 | 9.25 ± 2.65 | <0.001*** |
| Nasal-superior | 9.31 ± 3.15 | 8.81 ± 2.65 | 7.57 ± 1.76 | <0.001*** |
| Tempo | 10.79 ± 3.39 | 9.90 ± 2.50 | 8.77 ± 2.68 | <0.001*** |
| Macular | 13.24 ± 4.21 | 10.98 ± 3.01 | 8.81 ± 2.97 | <0.001*** |
| Optic disc | 10.70 ± 3.41 | 9.86 ± 2.77 | 8.57 ± 2.13 | <0.001*** |
| Tempo-inferior | 6.94 ± 2.74 | 6.55 ± 2.34 | 5.37 ± 1.98 | <0.001*** |
| Inferior | 8.26 ± 3.13 | 7.07 ± 2.22 | 6.09 ± 1.99 | <0.001*** |
| Nasal-inferior | 6.67 ± 2.20 | 5.86 ± 1.95 | 5.25 ± 1.54 | <0.001*** |
| CVV (×10^6^μm^3^) by grid | | | |  |
| Tempo-superior | 3854.42 ± 1161.55 | 3126.25 ± 1000.85 | 2781.94 ± 1325.85 | 0.008** |
| Superior | 5337.71 ± 1421.33 | 4103.50 ± 1180.82 | 3939.31 ± 1648.70 | 0.001** |
| Nasal-superior | 4498.04 ± 1180.26 | 3634.50 ± 1300.89 | 3111.44 ± 879.79 | 0.001** |
| Tempo | 4654.75 ± 948.52 | 3566.05 ± 1180.97 | 3729.50 ± 1461.04 | 0.002** |
| Macular | 5988.21 ± 1626.77 | 4212.88 ± 1526.14 | 4335.75 ± 1705.61 | <0.001*** |
| Optic disc | 4619.08 ± 1166.47 | 3917.10 ± 1592.17 | 3245.81 ± 860.01 | 0.009** |
| Tempo-inferior | 2984.63 ± 985.47 | 2541.85 ± 1050.30 | 2392.88 ± 786.13 | 0.121 |
| Inferior | 3630.75 ± 890.64 | 2552.33 ± 1497.19 | 2563.63 ± 1089.75 | 0.004** |
| Nasal-inferior | 2294.96 ± 658.37 | 1775.00 ± 998.40 | 1268.81 ± 421.21 | 0.001** |
| CSV (×10^6^μm^3^) by grid | | | |  |
| Tempo-superior | 6885.50 ± 965.58 | 6528.03 ± 1487.93 | 6144.81 ± 1085.50 | 0.201 |
| Superior | 9477.17 ± 1407.14 | 8292.60 ± 1778.57 | 7847.00 ± 1509.38 | 0.004** |
| Nasal-superior | 7709.33 ± 994.29 | 7338.90 ± 1905.14 | 6395.81 ± 771.02 | 0.027* |
| Tempo | 9160.54 ± 1006.69 | 8568.63 ± 1956.21 | 8345.63 ± 1531.64 | 0.242 |
| Macular | 10309.58 ± 1957.48 | 8343.23 ± 2257.90 | 7914.63 ± 1625.53 | <0.001*** |
| Optic disc | 8721.42 ± 1173.91 | 8394.38 ± 2083.74 | 7418.50 ± 910.74 | 0.052 |
| Tempo-inferior | 6515.17 ± 1042.61 | 6239.83 ± 1728.53 | 5993.88 ± 851.47 | 0.509 |
| Inferior | 7141.96 ± 931.40 | 7252.98 ± 5113.16 | 6105.50 ± 791.97 | 0.562 |
| Nasal-inferior | 5972.38 ± 675.90 | 5591.73 ± 1553.84 | 5055.81 ± 490.72 | 0.063 |
| CVI by grid | | | |  |
| Tempo-superior | 0.35 ± 0.05 | 0.32 ± 0.04 | 0.30 ± 0.07 | 0.004** |
| Superior | 0.36 ± 0.03 | 0.33 ± 0.04 | 0.32 ± 0.05 | 0.006** |
| Nasal-superior | 0.36 ± 0.03 | 0.33 ± 0.05 | 0.32 ± 0.04 | 0.004** |
| Tempo | 0.33 ± 0.04 | 0.29 ± 0.05 | 0.30 ± 0.05 | 0.002** |
| Macular | 0.36 ± 0.03 | 0.33 ± 0.05 | 0.34 ± 0.06 | 0.019* |
| Optic disc | 0.34 ± 0.04 | 0.31 ± 0.06 | 0.30 ± 0.04 | 0.015* |
| Tempo-inferior | 0.31 ± 0.05 | 0.28 ± 0.06 | 0.28 ± 0.05 | 0.107 |
| Inferior | 0.33 ± 0.04 | 0.26 ± 0.08 | 0.28 ± 0.07 | <0.001*** |
| Nasal-inferior | 0.27 ± 0.04 | 0.23 ± 0.07 | 0.20 ± 0.04 | <0.001*** |

Note: WF = Wide Field, LM = Low Myopia, MM = Moderate Myopia, HM = High Myopia, CVI = Choroidal Vascular Index, CV = Choroidal Volume, CVV = Choroidal Vascular Volume, CSV = Choroidal Stroma Volume, **P*<0.05, ***P*<0.01, and ****P*<0.001.

**Supplementary Figure**

**
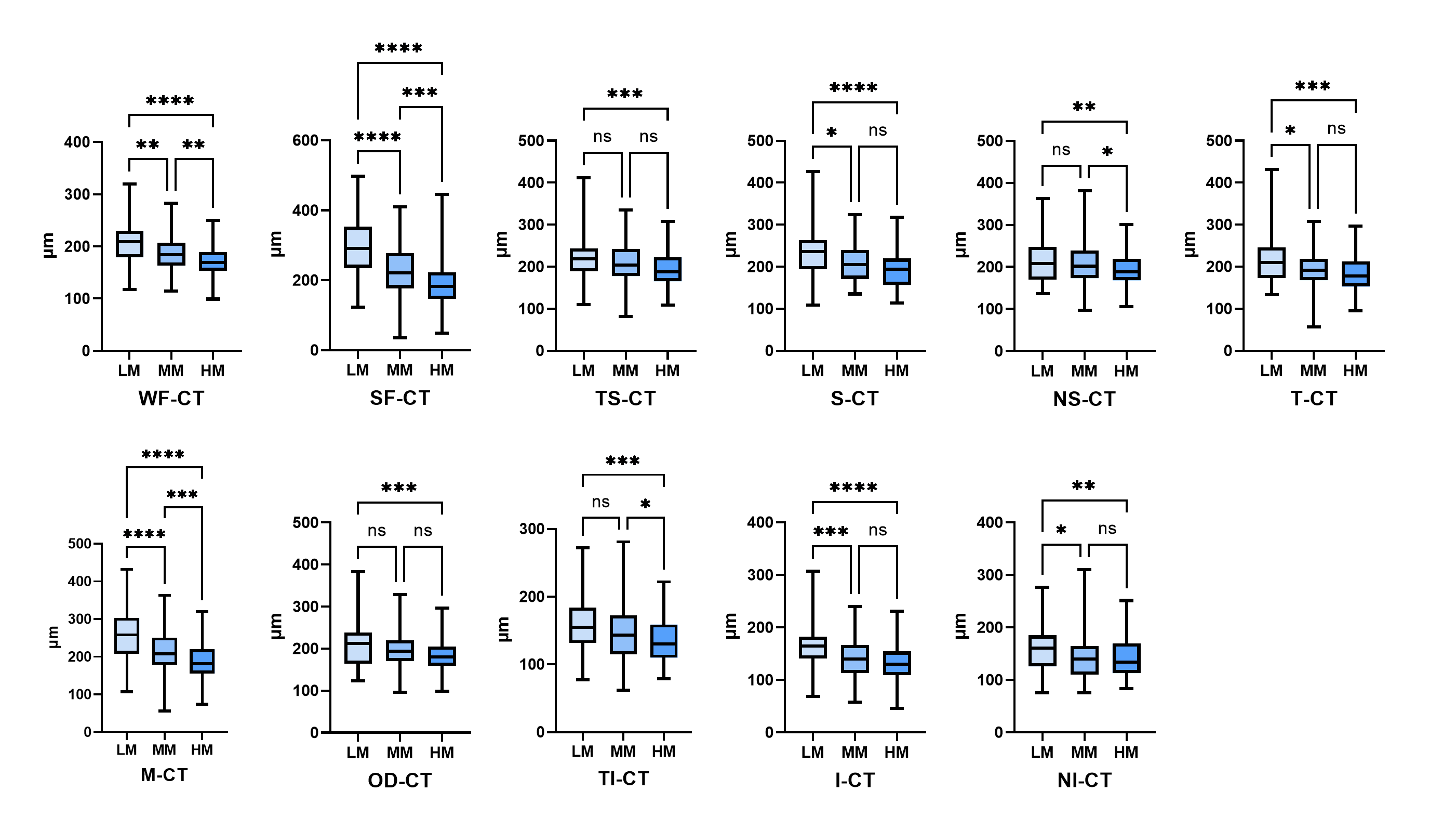
**

**Figure S1 The distribution of choroidal thickness in people with different degrees of myopia**

Note: LM = Low Myopia, MM = Moderate Myopia, HM = High Myopia, CT = Choroidal Thickness, WF = Wide-Field, SF = Sub-fovea, **P*<0.05, ***P*<0.01, ****P*<0.001, ****P*<0.001.

**
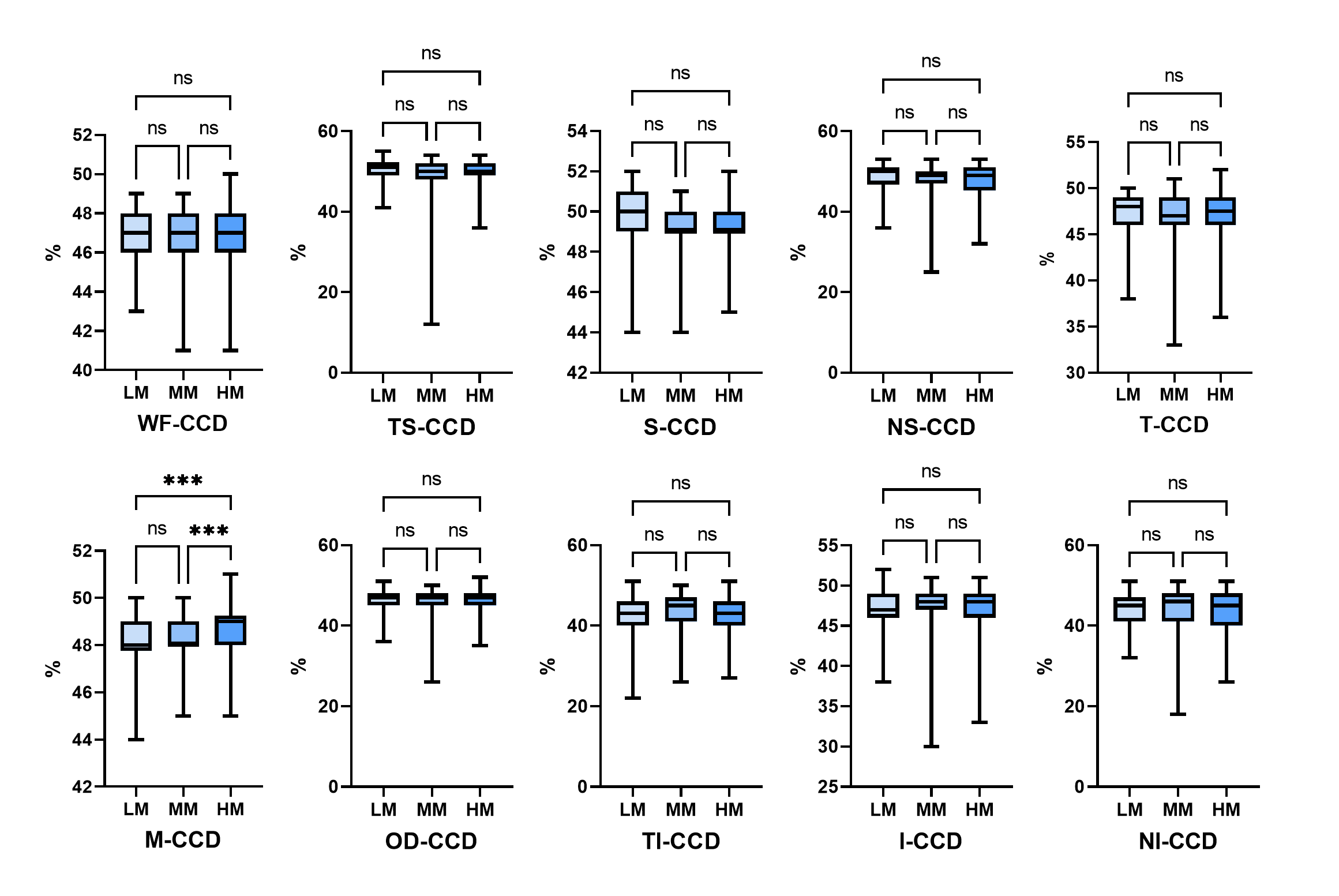
**

**Figure S2 Characteristics of choroidal capillary density in people with different degrees of myopia**

Note: LM = Low Myopia, MM = Moderate Myopia, HM = High Myopia, CCD = Choroidal Capillary Density, **P*<0.05, ***P*<0.01, ****P*<0.001, *****P*<0.0001.

**
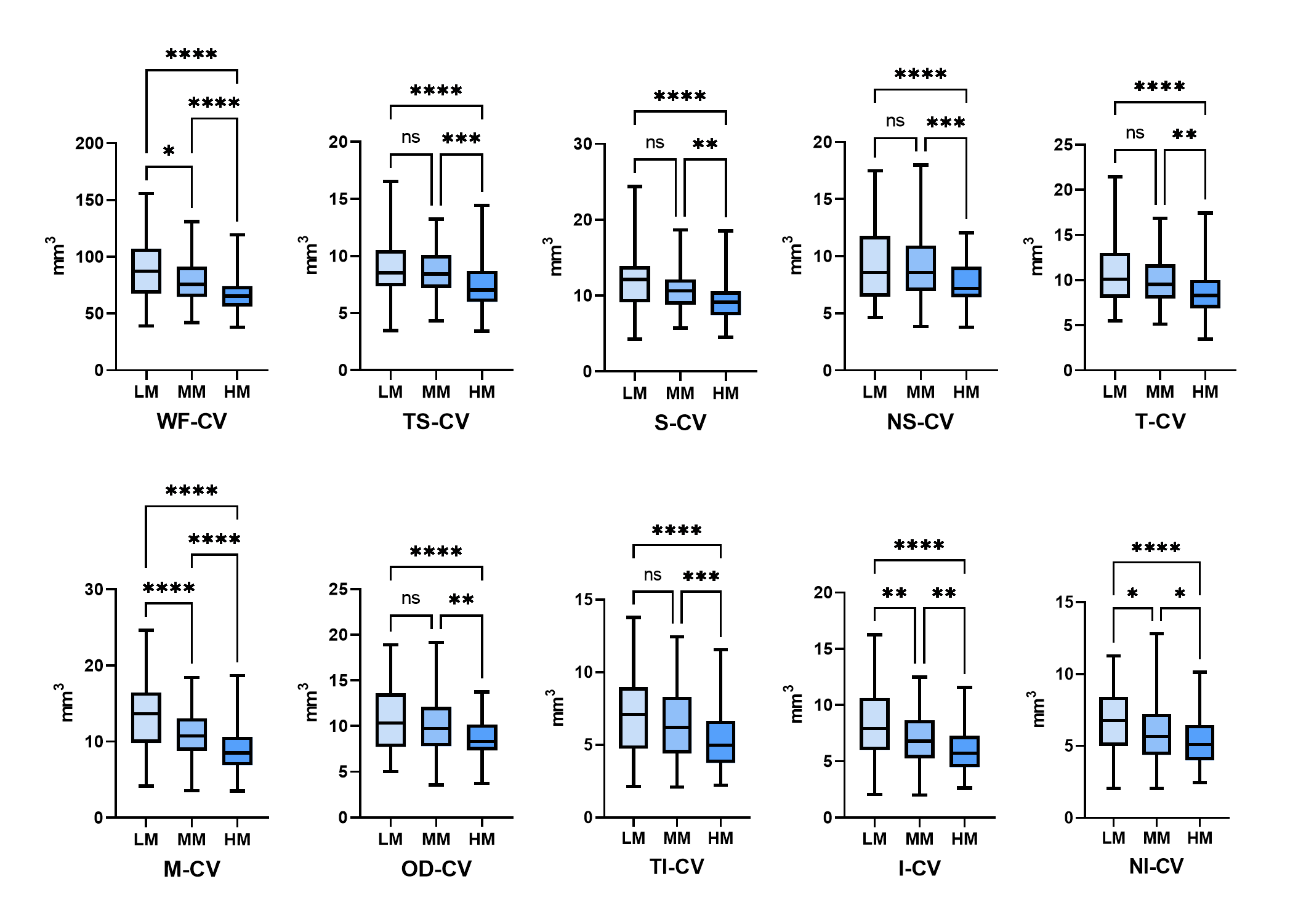
**

**Figure S3 Comparison of Choroidal Volume in Different Degrees of Myopia**

Note: LM = Low Myopia, MM = Moderate Myopia, HM = High Myopia, CV = Choroidal Volume, **P*<0.05, ***P*<0.01, ****P*<0.001, *****P*<0.0001.

**
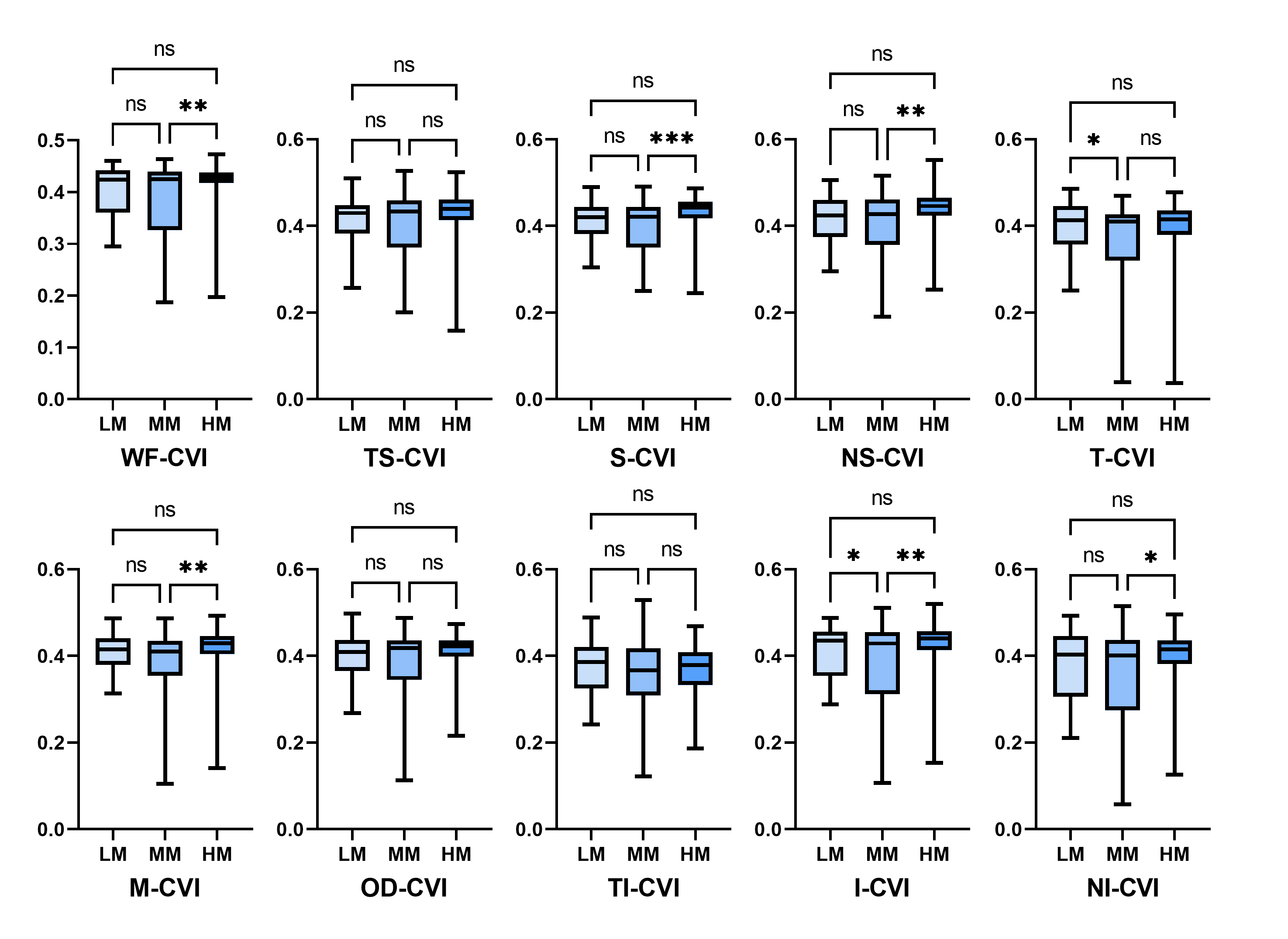
**

**Figure S4 Comparison of Choroidal Vascular Index in Different Degrees of Myopia**

Note: LM = Low Myopia, MM = Moderate Myopia, HM = High Myopia, CVI = Choroidal Vascular Index, **P*<0.05, ***P*<0.01, ****P*<0.001, *****P*<0.0001.
